# Using target enrichment sequencing to study the higher-level phylogeny of the largest lichen-forming fungi family: Parmeliaceae (Ascomycota)

**DOI:** 10.1186/s43008-020-00051-x

**Published:** 2020-12-14

**Authors:** Felix Grewe, Claudio Ametrano, Todd J. Widhelm, Steven Leavitt, Isabel Distefano, Wetchasart Polyiam, David Pizarro, Mats Wedin, Ana Crespo, Pradeep K. Divakar, H. Thorsten Lumbsch

**Affiliations:** 1 Science & Education, The Grainger Bioinformatics Center, Negaunee Integrative Research Center, Gantz Family Collections Center, and Pritzker Laboratory for Molecular Systematics, The Field Museum, 1400 S. Lake Shore Drive, Chicago, IL USA; 2 Department of Biology and M. L. Bean Life Science Museum, Brigham Young University, Provo, UT USA; 3 Lichen Research Unit, Biology Department, Faculty of Science, Ramkhamhaeng University, Ramkhamhaeng 24 Road, Bangkok, 10240 Thailand; 4 Departamento de Farmacología, Farmacognosia y Botánica, Facultad de Farmacia, Universidad Complutense de Madrid, 28040 Madrid, Spain; 5 Department of Botany, Swedish Museum of Natural History, PO Box 50007, SE-104 05 Stockholm, Sweden

**Keywords:** Next-generation sequencing, Target capture, HybPiper, Phylogenomics, Maximum likelihood, Bayesian interference, ASTRAL, *Parmotrema*, Parmelioideae, Protoparmelioideae

## Abstract

Parmeliaceae is the largest family of lichen-forming fungi with a worldwide distribution. We used a target enrichment data set and a qualitative selection method for 250 out of 350 genes to infer the phylogeny of the major clades in this family including 81 taxa, with both subfamilies and all seven major clades previously recognized in the subfamily Parmelioideae. The reduced genome-scale data set was analyzed using concatenated-based Bayesian inference and two different Maximum Likelihood analyses, and a coalescent-based species tree method. The resulting topology was strongly supported with the majority of nodes being fully supported in all three concatenated-based analyses. The two subfamilies and each of the seven major clades in Parmelioideae were strongly supported as monophyletic. In addition, most backbone relationships in the topology were recovered with high nodal support. The genus *Parmotrema* was found to be polyphyletic and consequently, it is suggested to accept the genus *Crespoa* to accommodate the species previously placed in *Parmotrema* subgen. *Crespoa.* This study demonstrates the power of reduced genome-scale data sets to resolve phylogenetic relationships with high support. Due to lower costs, target enrichment methods provide a promising avenue for phylogenetic studies including larger taxonomic/specimen sampling than whole genome data would allow.

## INTRODUCTION

Our understanding of evolutionary relationships of fungi at all phylogenetic levels has dramatically improved with the availability of genetic data from entire genomes following remarkable progress in sequencing technologies (Ametrano et al. 2019; Ebersberger et al. 2012; Robbertse et al. 2006; Spatafora et al. 2017). In addition to sequencing complete genomes, a number of more cost-efficient methods have been developed to sample subsets of genome-scale data. These include several direct sequencing approaches, such as restriction site associated DNA sequencing (RADseq) (Andrews et al. 2016), or capture sequencing approaches using baits, such as target enrichment of specific genes (Bragg et al. 2016) or ultra-conserved elements (Faircloth et al. 2012). These methods significantly reduce costs in comparison to sequencing entire genomes and thus will enable larger taxonomic or specimen sampling in comparative studies (Jones and Good 2016). RADseq has been used to address issues of delimitation and relationships of closely related Ascomycete species (Bracewell et al. 2018; Grewe et al. 2017; Grewe et al. 2018; Salas-Lizana and Oono 2018) and population biology (Talas and McDonald 2015). However, studies have shown that this approach is most appropriate for shallow systematics due to issues with homology at deeper evolutionary scales when genome sequences are more diverged (Harvey et al. 2016; Rubin et al. 2012).

Target enrichment sequencing particularly enhances genomic regions of interest within a heterogeneous mixture of DNA samples (i.e. metagenomes). For target enrichment sequencing, pre-designed RNA probes are added to the metagenomic DNA extracts and capture their complementary DNA sequences through hybridization. Hybridization concentrates the DNA of the targeted genomic regions and allows for selective next generation sequencing of these regions (Bragg et al. 2016; Mamanova et al. 2010). Therefore, target enrichment sequencing is a cost-effective sequencing approach compared to whole genome sequencing, which can be used for large taxonomic sampling (Dapprich et al. 2016). So far, target enrichment sequencing has not been widely used in Ascomycota, with notable exceptions including a study screening for pathogenicity genes (Alshuwaili et al. 2018) and another study understanding the impact of ancient hybridization in the diversification of a clade of lichenized fungi (Widhelm et al. 2019).

Parmeliaceae is the most diverse group of lichenized fungi with about 2800 currently accepted species (Jaklitsch et al. 2016) that underwent an increased diversification associated with the aridification during the Oligocene-Miocene transition (Kraichak et al. 2015). Within the family Parmeliaceae, two subfamilies are distinguished, Parmelioideae and Protoparmelioideae (Divakar et al. 2017), with the vast majority of species diversity occurring in Parmelioideae (Divakar et al. 2017). The family currently includes 69 accepted genera (Divakar et al. 2017). Previous studies suggest that the family originated during the Cretaceous and subsequently diversified after the Cretaceous-Paleogene (K-Pg) boundary (Huang et al. 2019). Speciation within genera mostly happened during the Miocene (Lumbsch 2016). The species of the family occur worldwide on all kinds of substrate and in all terrestrial ecosystems but have their centres of diversity in the tropics and temperate, winter rain areas (Crespo et al. 2010; Thell et al. 2012).

Parmelioideae includes the bulk of species in Parmeliaceae and consists of a number of strongly supported monophyletic clades (Crespo et al. 2010; Crespo et al. 2007; Divakar et al. 2015). Currently, this includes seven strongly supported major clades, all of which are included in this study. The clades are 1) alectorioid, 2) anzioid, 3) cetrarioid, 4) hypogymnioid, 5) parmelioid, 6) psiloparmelioid, and 7) usneoid. While previous multi-gene studies have shown the monophyly of these clades, the relationships among those remained largely unsupported. Recently, we have used 2556 genes sampled from whole genome sequences of 44 in-group taxa to study the evolutionary relationships among major clades in the subfamily Parmelioideae of Parmeliaceae (Pizarro et al. 2018). However, this study included a limited number of species due to the high costs of sequencing entire genomes and the computational burden of analysing thousands of genes (Pizarro et al. 2018). The smaller subfamily Protoparmelioideae currently includes three genera: the monotypic Australian *Maronina* Hafellner & R.W. Rogers, the pantropical *Neoprotoparmelia* Garima Singh, Lumbsch & I. Schmitt (dos Santos et al. 2019; Singh et al. 2018), which includes the majority of species previously included in *Maronina* s. lat., and the temperate *Protoparmelia* M. Choisy (Poelt and Grube 1992).

The main focus of this study was to assess the phylogenetic potential of cost-effective target enrichment sequencing approach using the hyper-diverse family Parmeliaceae as a model system. Our specific objectives were: 1) test our recent phylogenetic hypotheses based on multi-gene and whole genome data sets, 2) address phylogenetic relationships of major clades in Parmelioideae, and 3) test the power of target enrichment data sets to resolve phylogenetic relationships in ascomycetes, using Parmeliaceae as an example. We have augmented this taxon sampling to include a total of 81 in-group taxa including samples from both Parmelioideae and the other subfamily of Parmeliaceae, Protoparmelioideae (Divakar et al. 2017). We compare results from a target enrichment dataset with gene extraction methods from whole genome assemblies. Ultimately, we selected the best results of all gene extraction methods to produce a robust phylogenetic tree of all taxa.

## MATERIALS AND METHODS

### Taxon sampling

We included 81 representatives of lichen-forming fungal species from Parmeliaceae and five outgroup species in this phylogenomic study (Supplementary Table 1). Seventy-eight samples were selected to represent the seven major clades in subfamily Parmelioideae and three samples represented the subfamily Protoparmelioideae (Divakar et al. 2017; Divakar et al. 2015). Sequences from five additional species (*Arthonia rubrocincta* G. Merr. ex Lendemer & Grube, *Cladonia uncialis* (L.) Weber ex F.H. Wigg., *Lobaria pulmonaria* (L.) Hoffm., *Rhizoplaca melanophthalma* (DC.) Leuckert, and *Umbilicaria pustulata* (L.) Hoffm.) were selected as outgroups.

### Target enrichment and sequencing

Baits design and target enrichment protocols were adopted from an earlier study (Widhelm et al. 2019). In short, 400 gene sequences were selected from the genome of *Lobaria pulmonaria* (available at JGI) and from a transcriptome assembly of *Evernia prunastri* [transcriptome sequence data published in (Meiser et al. 2017)]. The introns of the selected gene sequences were masked by aligning them with transcriptome assemblies of *Pseudevernia furfuracea* and *Lasallia pustulata* (Meiser et al. 2017), then all masked gene sequences were sent to Arbor Biosciences (Ann Arbor, MI, USA) for the RNA baits production.

The DNA of Parmeliaceae species was isolated with the ZR Fungal/Bacterial DNA MiniPrep according to the manufacturer’s protocol (Zymo Research, Irvine, CA, USA). The isolated DNA was converted into libraries with the KAPA Hyper Prep Kit (KAPA Biosciences, Wilmington, MA, USA) using the Adapterama dual-indexing system to uniquely barcode all samples (Widhelm et al. 2019). A pool of all libraries was enriched for the 400 targeted genes by hybridization with the RNA baits. Enriched libraries were then paired-end sequenced with the Illumina MiSeq sequencer at the Field Museum’s Pritzker Laboratory using the MiSeq 600-cycle sequencing kit version 3 (Illumina, San Diego, CA, USA).

In addition to target enrichment, we used sequences from nine Parmeliaceae species for which we had the whole genome sequenced: *Everniopsis trulla* (Ach.) Nyl.*, Psiloparmelia denotata* Elix & T.H. Nash*, Usnea aurantiacoatra* (Jacq.) Bory*,* and six *Xanthoparmelia* species. For all species, DNA was isolated using the ZR Fungal/Bacterial DNA MiniPrep according to the manufacturer’s protocol (Zymo Research, Irvine, CA, USA) – the same kit that was used to isolate DNA for target enrichment. Library construction and sequencing were done at the DNA services facility of the University of Illinois at Urbana-Champaign as described previously (Pizarro et al. 2018).

### Gene extraction from target enrichment, whole genome sequencing data, and de novo assemblies

We used a combination of newly sequenced target enrichment data, newly sequenced whole genome data, and existing whole genome data for the target gene assembly and extraction. The Illumina MiSeq target enrichment sequencing result and Illumina whole genome sequencing results were demultiplexed in BaseSpace (Illumina) before being downloaded as raw reads to a local server. In addition, we downloaded all available whole genome sequencing data of earlier phylogenomic analyses (Abdel-Hameed et al. 2016; Greshake et al. 2016; Grewe et al. 2017; Grewe et al. 2018; Leavitt et al. 2016; McDonald et al. 2013; Meiser et al. 2017; Pizarro et al. 2018). All raw reads were trimmed with Trimmomatic v0.33 (Bolger et al. 2014), setting a quality threshold of 10 (LEADING:10 TRAILING:10) and a minimum read length of 25 bp (MINLEN:25). The surviving paired-end reads were used in HybPiper (Johnson et al. 2016) for gene assembly and extraction based on a target gene file. We generated this target gene file for HybPiper from the genome assembly of the Parmeliaceae species *P. furfuracea* (Meiser et al. 2017). We used BLASTn to search all gene models of the *P. furfuracea* genome with the bait sequence file as a query and identified 355 full-length genes. We extracted the complete exons of the identified *P. furfuracea* genes and used these sequences as a target gene file for all further gene extractions in this study. We translated the extracted nucleotide sequences into amino acid sequences since HybPiper did perform better using the BLASTx option, which uses an amino acid target gene file, instead of the BWA option, which uses a nucleotide target gene file. We then used the translated target gene file with the HybPiper wrapper script ‘reads_first.py’ on all trimmed Illumina sequence reads. HybPiper created one file folder for each species containing all assembly data and gene sequences, which we stored for further evaluation. All files that were derived from target enrichment data were tagged with “_target”. Depending on the type of input sequence data, we named both methods as either “target enrichment method” or “whole genome method”.

In exploratory analyses where samples prepared with the “whole genome method” recovered relatively fewer loci with the HybPiper pipeline, we subsequently assembled the whole genome sequence reads into de novo draft genomes prior to a target gene identification with Exonerate (Slater and Birney 2005) – we named this approach “de novo assembly method”. De novo assemblies of the trimmed paired-end Illumina reads were constructed with SPAdes v3.5 or v3.11 (Bankevich et al. 2012). All assemblies were processed by the HybPiper script ‘exonerate_hits.py’ (part of the HybPiper wrapper) and the amino acid target gene file. All extracted target gene regions were transformed into the HybPiper wrapper output format with one file folder for each species and the different gene sequences separated in subfolders. The file names that resulted from the “de novo assembly method” were tagged with “_spades.”

The combined output from “target enrichment,” “whole genome,” and “de novo assembly methods” was evaluated and visualized with the HybPiper scripts ‘get_seq_lengths.py’ and ‘gene_recovery_heatmap.py.’ The scripts provided an overview about sequencing and assembly success and allowed a direct comparison of the results of the “whole genome method” and the “de novo assembly method,” which were both based on the same input data. We evaluated the sequencing success of each method by comparing the total number of recovered amino acids and the average length of recovered gene sequences. For comparative purposes, we visualized the results of the three methods in a heatmap using the script ‘gene_recovery_heatmap.py.’ We then used the script ‘retrieve_sequences.py’ to save the nucleotide and amino acid sequences of the best performing method for each species in a fasta file. We removed five fasta files of genes which contained only sequences of 50% or less of all taxa (i.e. less than 43 sequences), leaving 350 gene files for further analyses.

### Selection of most informative gene sequences

We first aligned all 350 gene files with Mafft v7.450 (Katoh and Standley 2013) and removed ambiguously aligned regions from each alignment using Gblocks v0.91 (Castresana 2000; Talavera and Castresana 2007) using relaxed parameter settings (b2 = half + 1, b4 = 5, b5 = half). We calculated the substitution rate for each gene with baseml implemented in PAML v4.9e (Yang 1997). To infer comparable substitution rates for all genes, we used the same constrained tree for each gene analysis. For the constrained tree, we concatenated all gene alignments with FASconCAT-G (Kueck and Longo 2014) and inferred the Maximum Likelihood tree with RAxML v8.2.11 (Stamatakis 2014) using the GTR + G model. We rooted the resulting tree with *Arthonia rubrocinta* and pruned it using the Python library DendroPy (Sukumaran and Holder 2010) to match the taxa of each gene alignment. The constrained tree was marked at 108 Ma for the origin of Parmeliaceae (Amo de Paz et al. 2011) prior to its use with each gene alignment for branch length and absolute substitution rate calculation in baseml (clock = 1; model = 7). Two genes showed extraordinarily high rates and hence were excluded. All other genes were sorted based on their substitution rate estimates from the slowest to the fastest evolving genes (Supplementary Table 2). We then built a concatenated alignment of the ten slowest evolving genes, then progressively increased the alignment stepwise with the next 10 faster evolving genes until 340 genes were included. We used all resulting 34 multigene alignments to calculate a Maximum Likelihood tree with RAxML v8.2.11 using the GTR + G model and the fast bootstrap option. We then calculated the average bootstrap value for each tree to identify the gene set that produced the best-supported phylogeny. The genes of this phylogeny were selected for all subsequent phylogenomic analyses. In addition, the remaining fast-evolving genes were used for separate phylogenomic analyses to identify potential conflicting phylogenetic signals.

### Phylogenomic analyses

Evolutionary relationships were estimated from the concatenated alignment of 250 selected genes using Maximum Likelihood (ML) and Bayesian interference (BI). ML trees were estimated with the programs RAxML and IQ-TREE (Nguyen et al. 2015) using the GTR + G model for the nucleotide data set, which was partitioned by genes. For each RAxML analysis 100 bootstrap replicates and for each IQ-TREE analysis 1000 bootstrap replicates were calculated using the fast bootstrapping option implemented in RAxML and IQ-TREE, respectively. BI trees were calculated with MrBayes v3.2.6 (Huelsenbeck and Ronquist 2001; Ronquist et al. 2012) using the GTR + G model for the nucleotide data set, which was partitioned by genes. For this analysis, two runs (four chains) with 1,500,000 iterations each were performed in parallel. Trees were sampled every 500 generations from the posterior distribution, and the first 25% of all sampled trees were discarded as the burn-in. We ensured convergence by a resulting ‘average standard deviation of split frequencies’ lower than 0.0000001 and ‘Effective Sample Size’ values higher than 200 in TRACER (Rambaut and Drummond 2007).

In addition to the concatenated-based phylogenies, we used coalescent-based methods to estimate a species tree given the individual gene trees of the 250 selected genes. All selected gene sequences were aligned with Mafft v7.450 (Katoh and Standley 2013) and trimmed with Gblocks v0.91 using relaxed parameter settings (b2 = half + 1, b4 = 5, b5 = half). ML trees of each gene were calculated with IQ-TREE using the GTR + G model. For each analysis 1000 bootstrap replicates were calculated using the fast bootstrapping option implemented in IQ-TREE. We contracted low support branches (bootstrap < 20) of all individual trees using Newick Utilities (Junier and Zdobnov 2010) before using all trees as input for a coalescent-based species tree estimation with ASTRAL-III (Zhang et al. 2018).

In addition to the selected 250 slow-evolving genes, we concatenated all remaining fast-evolving gene sequences to estimate their phylogenetic relationship. The concatenated matrix was partitioned by genes and used for ML analyses with the programs RAxML and IQ-Tree using the GTR + G model. For each RAxML analysis 100 bootstrap replicates and for each IQ-TREE analysis 1000 bootstrap replicates were calculated using the fast bootstrapping option implemented in RAxML and IQ-TREE, respectively. All resulting phylogenetic trees were drawn with the program FigTree v1.4.2 (Rambaut 2009).

### Availability of data and material

The sequences produced in this paper have been deposited in the NCBI Sequence Read Archive (SRA) with the accession numbers SRR13125477, SRR1315498, SRR13125762, SRR13167197, SRR13125985, SRR13126647, SRR13126796, SRR13126828, SRR13126859. Target enrichment gene set and multiple sequence alignments are available at FigShare DOI:10.6084/m9.figshare.13238453.

## RESULTS AND DISCUSSION

### Phylogenomic data

The three different methods that were used to recover the targeted genes in Parmeliaceae executed with different success. While the “target enrichment method” used RNA baits for target capturing prior to sequencing, the “whole genome method” and “de novo assembly method” used the same whole genome sequence data either as raw data input or assembled into draft genomes to capture the targeted genes. The average length of all recovered target genes was 82,196 (SD = 53,643) amino acids for the “whole genome method” compared to 138,069 (SD = 10,845) amino acids for the “de novo assembly method” (Supplementary Table 3). Therefore, the recovery of the target genes was comparatively improved with the “de novo assembly method,” in particular for species with low sequence coverage. The “target enrichment method” recovered a total of 123,788 (SD = 9578) amino acids, which performed better than the “whole genome method”, but the “de novo assembly method” recovered on average slightly more amino acids. The strength of each method is also visible in the heatmap showing the recovery of all genes for each species (Supplementary Figure 1). The “de novo assembly method” was more successful than the “whole genome method,” which performed weakly for many species and even completely failed for some species. The heatmap further indicated that the “target enrichment method” failed for some gene sequences (30 genes were recovered by only half of their length or less). Since many of these genes were successfully recovered with the other two methods using whole genome sequencing data, it might be a consequence of the target enrichment procedure and indicate that some baits failed to capture the Parmeliaceae gene sequences.

**Fig. 1:**
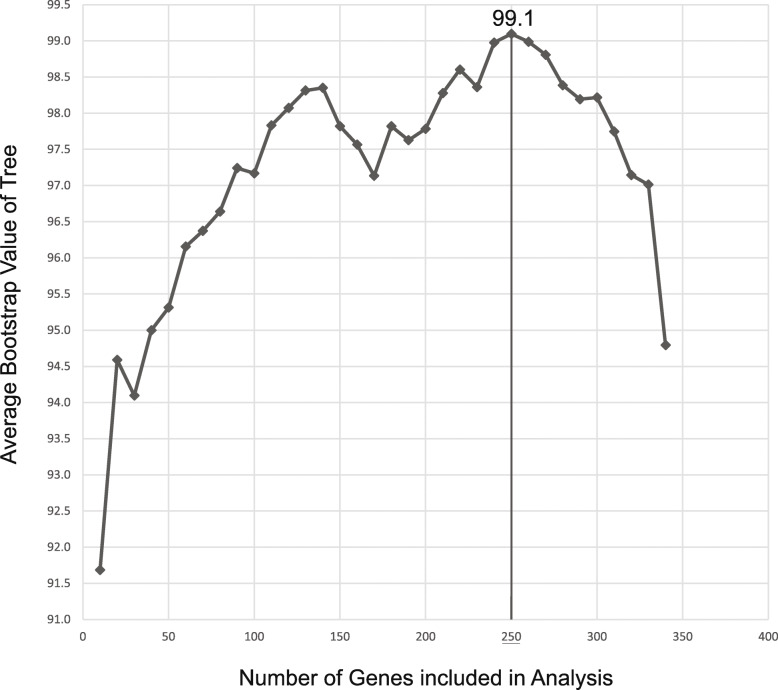
Correlation of the number of concatenated genes and the average bootstrap value of the resulting phylogenetic tree. The phylogenetic tree of each data point was reconstructed with the concatenated alignment of the selected genes with RAxML using the GTR + G model. Bootstrap values were calculated with the fast bootstrapping option in RAxML

The most informative subset of genes for phylogenomic analyses was identified by the maximum number of slowest evolving genes that reconstructed the best supported phylogenetic tree. We created 34 multigene matrices that included concatenated alignments from 10 to 340 genes. All resulting phylogenetic trees had average bootstrap values above 91 (Fig. 1). Including faster evolving genes in the multigene matrix had an overall positive effect on the phylogenetic tree reconstruction until we reached 250 genes, with a maximum average bootstrap value at 99.1. Remarkably, after 250 genes, the addition of more (faster evolving) genes to the multigene matrix decreased the average bootstrap of the trees. Therefore, we selected the 250 genes that achieved the maximum average bootstrap value for all subsequent phylogenetic inferences. The final nucleotide multigene matrix had a dimension of 86 taxa and 270,160 characters and 178,588 distinct alignment patterns with 3.37% of undetermined characters or gaps.

### Phylogenetic relationships

**Fig. 2:**
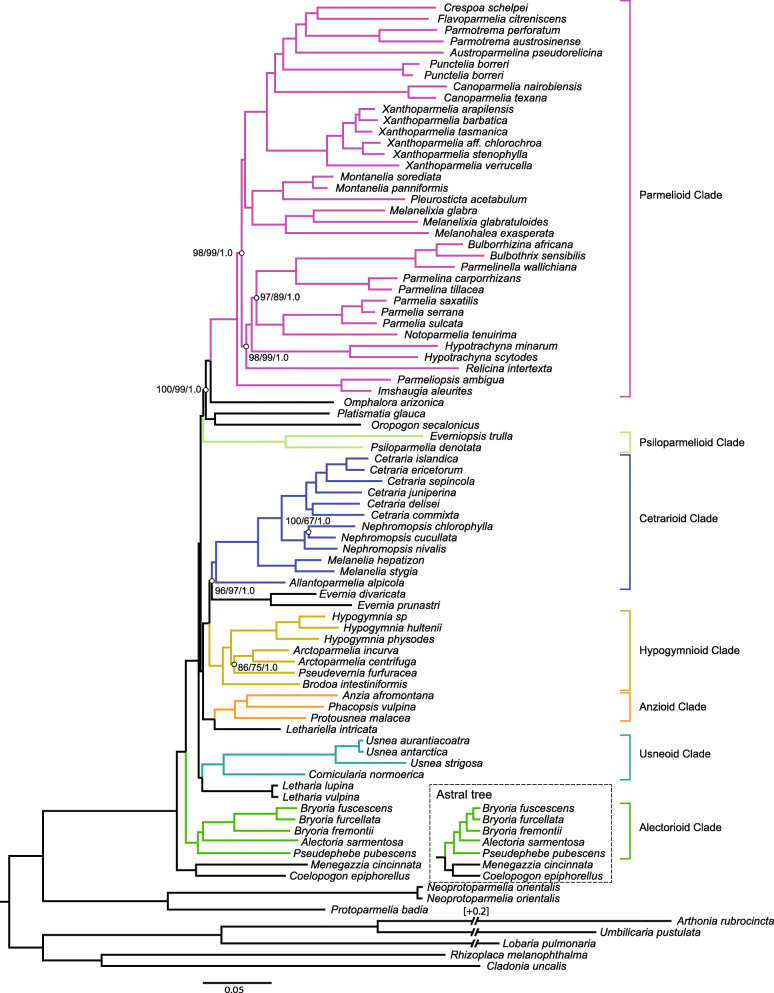
Phylogenetic relationships among major lineages of Parmeliaceae, represented by 81 specimens. The tree shown was generated by Maximum Likelihood inference using IQ-TREE of a data set containing the 250 most phylogenetic informative genes of the target enrichment gene set. Additional trees generated by Maximum Likelihood using RAxML and Bayesian interference using MrBayes resulted with the same topology. All nodes of the three trees received 100% bootstrap support (BS) with IQ-TREE and RAxML or 1.0 posterior probability (PP) with MrBayes unless highlighted by an open circle and the respective support values (IQ-TREE BS/RAxML BS/MrBayes PP). Major clades of Parmeliaceae are highlighted by different colors. The dotted box highlights the conflict between the concatenated-based trees (IQ-TREE, RAxML, MrBayes) and coalescent-based tree (ASTRAL-III). The unit of branch length is substitutions per site

Phylogenies inferred from the concatenated target enrichment dataset under Maximum Likelihood and Bayesian inference recovered identical topologies and hence only the IQ-TREE tree is shown here (Fig. 2). The coalescent-based ASTRAL-III tree conflicted in one node altering the placement of the *Coelopogon/Menegazzia* clade (Fig. 2, Supplementary Figure 2). All nodes in the Bayesian analysis received a posterior probability of 1.0, whereas five nodes in the IQ-TREE analysis and nine nodes in the RaxML analysis received bootstrap support of less than 100% (Fig. 2, Supplementary Figure 2). However, with the exception of the low supported sister-group relationships of *Arctoparmelia* and *Pseudevernia* (86% in the IQ-TREE and 75% in the RAxML analyses), of a clade consisting of the genera *Notoparmelia + Parmelia* and a clade including the genera *Bulborrhizina, Bulbothrix, Parmelinella,* and *Parmelina* (89% in the RAxML tree), and of *Nephromopsis chlorophylla* and *N. cucullata* (67% in the RAxML analysis), the bootstrap support for those was above 95% and is considered here to be strong. The monophyly of the two subfamilies Protoparmelioideae and Parmelioideae was strongly supported. The phylogenetic tree of all remaining fast-evolving genes resulted in a similar topology as the tree of the 250 slow-evolving genes, with the exception of a strongly supported cluster of *Omphalora arizonica, Everniopsis trulla, Psiloparmelia denotata, Oropogon secalonicus,* and *Platismatia glauca* (Supplementary Figure 2)*.* Therefore, some of the fast-evolving genes may reflect a different evolutionary history of these taxa than the 250 selected genes. In addition, many relationships in the phylogenetic inference of the fast-evolving genes were either unresolved or less supported indicated by poor bootstrap support. The different evolutionary history and the lack of phylogenetic signal of some fast-evolving genes may have caused the decrease of the average bootstrap support when more fast-evolving genes were included to the 250-gene phylogeny (Fig. 1).

The overall phylogeny of the 250 selected genes was similar to topologies inferred from a multi-gene data set with a larger taxon sampling (274 ingroup taxa) (Divakar et al. 2015) and a genomic data set with a smaller taxon sampling (44 ingroup taxa) (Pizarro et al. 2018). Here we focus on describing the phylogenetic relationships of major clades and differences to either of these previous studies. The phylogenetic position of the *Coelopogon/Menegazzia* clade was unresolved in previous studies, being either sister to the usneoid clade without support (Divakar et al. 2015), sister to the alectorioid clade, or sister to all remaining Parmelioideae – again without support (Pizarro et al. 2018). In our concatenated-based analyses, the predominantly southern Hemisphere *Coelopogon/Menegazzia* clade was an early divergent lineage within the subfamily Parmelioideae, as it formed a strongly supported sister-group relationship with all remaining Parmelioideae (Fig. 2, Supplementary Figure 2). However, in the coalescent-based analysis, the *Coelopogon/Menegazzia* clade was sister to the alectorioid clade (Fig. 2, Supplementary Figure 2). An inconsistent placement of *Menegazzia* depending on the use of concatenated or coalescent-based analyses was also documented in an earlier study on Parmeliaceae (Pizarro et al. 2018). In our study, we added *Coelopogon* as a sister taxon to *Menegazzia* to resolve the placement of *Coelopogon/Menegazzia*; however, the phylogenetic position of the two taxa remained conflicting between the two analyses. Concatenation-based analyses have been shown to overestimate phylogenetic relationships in large datasets (Edwards et al. 2007). Furthermore, coalescent-based analyses can be inconsistent under gene flow and incomplete lineage sorting (Solis-Lemus and Ane 2016; Solis-Lemus et al. 2016) and when individual gene trees are erroneous due to evolutionary rate heterogeneity (Koch et al. 2018). In this study, we particularly selected slow-evolving genes, which reduces the evolutionary rate heterogeneity of the used genes. However, the position of *Coelopogon/Menegazzia* remained in conflict whether coalescent-based or concatenated-based analyses were used (Fig. 2).

The alectorioid clade was resolved as monophyletic and was sister to the remaining Parmelioideae, which agreed with the placement in the ML tree of the genomic analysis of Pizarro et al. (2018). In contrast to Krog’s hypothesis (Krog 1982), *Letharia* and *Lethariella* did not form a sister-group relationship in our study (Fig. 2). The genus *Letharia* was only included in Divakar et al. (2015) but its phylogenetic relationships remained unresolved. In our study, the two *Letharia* species formed a strongly supported sister-group to the usneoid clade, which was also strongly supported as monophyletic. Our results are in accordance with the phenotypic similarities of *Letharia* with usneoid lichens (Crespo et al. 2007; Krog 1982). The anzioid clade formed a strongly supported sister-group with *Lethariella intricata* (Moris) Krog, the type species of the genus. Previously, *Lethariella* clustered with *Letharia* (Divakar et al. 2015) but in that study, two other species of the genus, *L. cashmeriana* Krog and *L. togashii* (Asahina) Krog were included and which could indicate that the genus is not monophyletic. The anzioid clade+*Lethariella* formed a strongly supported sister-group relationship to a clade including the hypogymnioid and cetrarioid clades and the genus *Evernia.* The relationships of *Evernia* and the cetrarioid clade was also strongly supported in a previous study (Pizarro et al. 2018) but lacked support in another (Divakar et al. 2015). Also, the strongly supported relationships of the hypogymnioid clade with the cetrarioid clade+*Evernia* has been found previously (Pizarro et al. 2018). The psiloparmelioid clade that was not included in Pizarro et al. (2018) had an unsupported placement in Divakar et al. (2015). In our study it formed a strongly supported sister-group to a clade that includes the parmelioid clade (including the early diverging clade consisting of *Imshaugia* and *Parmeliopsis*), the monotypic genus *Omphalora,* and a clade including *Oropogon* and *Platismatia*. This agrees with the tree topology in Pizarro et al. (2018) but it lacked support in that analysis. All genera for which more than one species was included formed strongly supported, monophyletic groups with the exception of the genus *Parmotrema. Parmotrema schelpei* (Hale) D. Hawksw., which was placed in the subgenus *Crespoa* by some authors (Hawksworth 2011; Kirika et al. 2016) does not cluster with the two other species of *Parmotrema* included in this study. This supports the segregation of the clade at the generic level and the acceptance of the genus *Crespoa* (Lendemer and Hodkinson 2012). In previous studies the relationship of *Pleurosticta* was unresolved (Crespo et al. 2010; Divakar et al. 2012) and it was grouped with species corresponding to the genus *Montanelia* (Crespo et al. 2010; Divakar et al. 2012; Leavitt et al. 2015) In our study, *Pleurostictca* formed an independent lineage that was the strongly supported as sister-group to *Montanelia*.

### Utility of target capture data sets to resolve phylogenetic relationships

The Parmeliaceae phylogenies include 35 taxa that were sequenced by target enrichment, together with 46 other ingroup taxa for these whole genomes were sequenced. A comparison of target enrichment to whole-genome sequencing or multi-gene sequencing of earlier Parmeliaceae studies revealed the limits of both whole-genome and multi-gene methods. Multi-gene phylogenies are affordable and allow the inclusion of many taxa, but they can be limited on the amount of phylogenetic information (Crespo et al. 2007; Divakar et al. 2017; Divakar et al. 2015). In comparison, whole genomes provide thousands of gene sequences for phylogenetic analyses, but the high sequencing cost and computational burden can limit the number of taxa (Pizarro et al. 2018). Target enrichment sequencing overcomes the limits of both whole-genome and multi-gene sequencing methods, since it allows for the affordable sequencing of hundreds of genes of multiple taxa, all can be pooled together in one sequencing run. This method specifically enriches targeted gene sequences prior to next-generation sequencing, which reduces sequencing costs and has the additional benefit of sorting symbiotic metagenomes, such as whole lichen DNA isolates. The enrichment of genes also works well when only low amounts of DNA are available and therefore qualifies as a method for rare species and older herbarium specimens, for which traditional molecular methods would fail. As a reduced genome representation method, target enrichment usually recovers fewer genes than whole genome sequencing, however a well-chosen gene set might improve phylogenies and outperform large genomic datasets, as shown by the selection of the most phylogenetically informative 250 genes out of 350 genes to reconstruct a remarkably well supported phylogeny for Parmeliaceae (Fig. 1). In the only other phylogenomic study using target enrichment data in ascomycetes so far, patterns consistent with ancient hybridization could be detected (Widhelm et al. 2019). This demonstrated that, in addition to reconstructing phylogenies, these data sets are also powerful in identifying historical processes shaping diversity of organisms.

The target enrichment method can reach its limits when the baits sequences differ too much from the target sequences, hence different baits sets are required for diverse taxon samplings. To overcome this limitation, a universal bait set of 353 conserved flowering plant genes was developed and is publicly available for phylogenomic analyses of flowering plants (Brewer et al. 2019; Johnson et al. 2019). A similar universal bait set could be optimized for fungal groups and take over as the next generation of phylogenetic marker genes. Hence, target enrichment sequencing provides a powerful novel avenue with great potential for the future of fungal phylogenomic research.

## CONCLUSION

For phylogenomic analyses, target enrichment sequencing represents an effective and inexpensive alternative for sequencing hundreds of genes when compared to other methods such as whole genome sequencing. We used target enrichment sequencing to generate data for the phylogenetic reconstruction of the largest family of lichen-forming fungi: Parmeliaceae. All sequenced genes were filtered for the 250 most informative genes. These genes were implemented in coalescent-based and concatenated-based phylogenetic applications, which estimated highly-supported phylogenetic trees. The trees of both methods differed in the placement of *Menegazzia*, potentially due to misleading effects of incomplete lineage sorting or evolutionary rate heterogeneity. While future phylogenomic methods might help to address these issues in phylogenetic reconstructions of large datasets, this study highlights the advantages that target enrichment has for fungal research. The inexpensive and straight-forward target enrichment sequencing approach will open new possibilities for fungal researchers to incorporate the use of large genomic data sets into their research.

## Supplementary Information

**Additional file 1 **: **Supplementary Figure 1.** Heatmap summarizing the results of target gene recovery. Each field represents the percentage of recovered amino acids of every gene for a respective taxon. All taxa were sorted by the methods that was used for the gene recovery.

**Additional file 2 **: **Supplementary Figure 2.** Phylogenetic relationships among major lineages of Parmeliaceae. Four trees were generated by Maximum Likelihood inference using a) IQ-TREE, b) RAxML, c) Bayesian interference using MrBayes, or d) a coalescent-based species tree calculation of a data set containing the 250 most phylogenetic informative genes of the target enrichment gene set. In addition, two trees were generated by Maximum Likelihood inference using e) IQ-Tree and f) RAxML of data containing the 89 fastest evolving genes. Numbers at tree branches represents IQ-TREE bootstrap, RAxML bootstrap, or MrBayes posterior probability values, respectively. The unit of branch lengths of the IQ-TREE, RAxML, and MrBayes trees is substitutions per site. Branch lengths of the tree generated by ASTRAL-III are in coalescent units.

**Additional file 3 **: **Supplementary Table 1.** Collection details of taxa used in this study.

**Additional file 4 **: **Supplementary Table 2.** Absolute substitution rates calculated by baseml using the GTR model; the unit of rates is substitutions per site per million years.

**Additional file 5 **: **Supplementary Table 3.** Results of target gene recovery using the “target enrichment method”, the “whole genome method”, or the “de novo assembly method”.
