# Supplementary figures and images for: Using target enrichment sequencing to study the higher-level phylogeny of the largest lichen-forming fungi family: Parmeliaceae (Ascomycota)
Source: IMA Fungus. 2020 Dec 14;11:27. doi: 10.1186/s43008-020-00051-x (PMC7734834; doi:10.1186/s43008-020-00051-x)

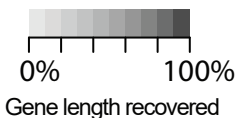[illegible]

target enrichment m.

Supplement: Supplementary file 1 — Additional file 1 : Supplementary Figure 1. Heatmap summarizing the results of target gene recovery. Each field represents the percentage of recovered amino acids of every gene for a respective taxon. All taxa were sorted by the methods that was used for the gene recovery. [file 43008_2020_51_MOESM1_ESM.pdf]

a) IQ-TREE

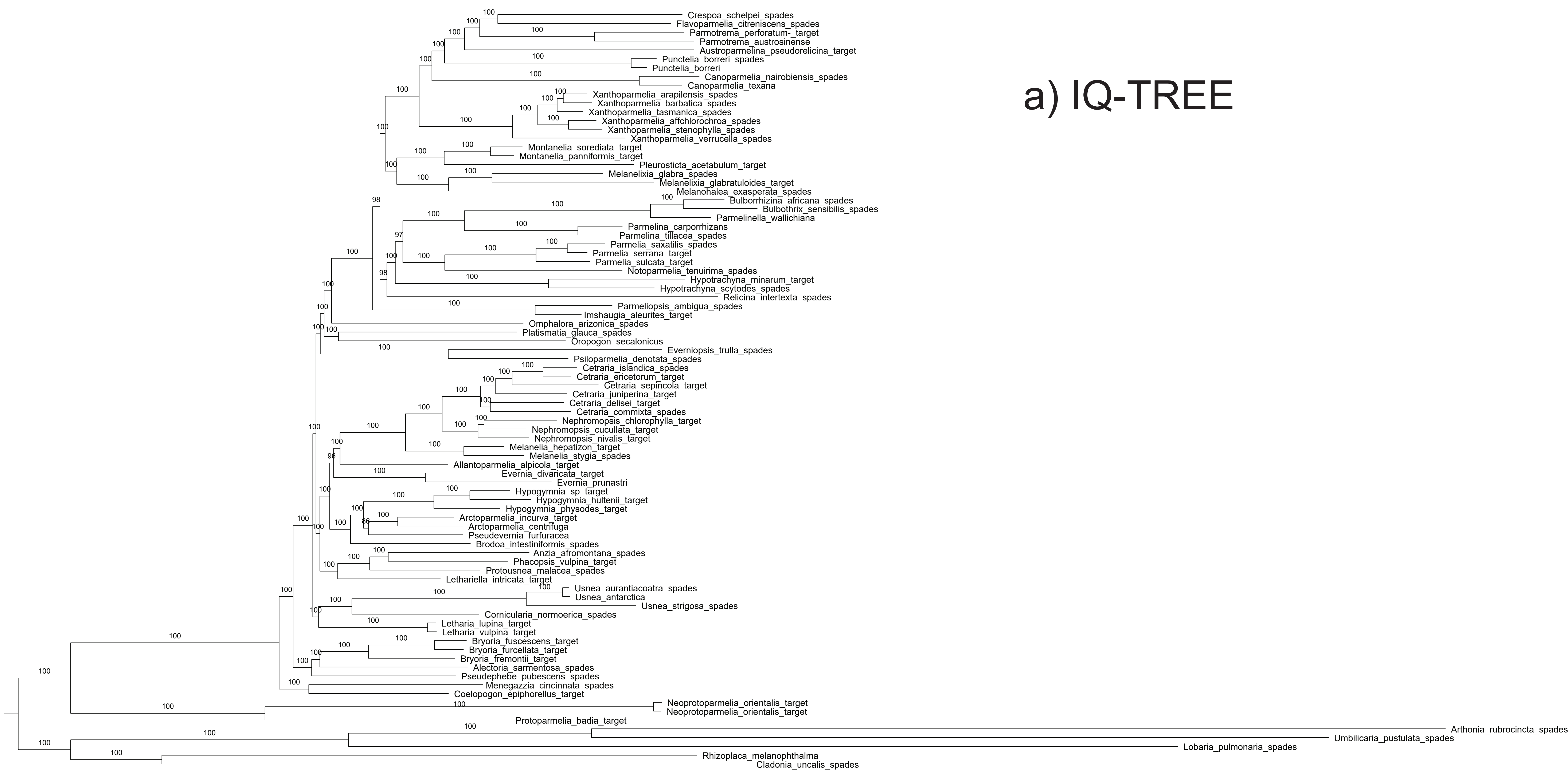

b) RAxML

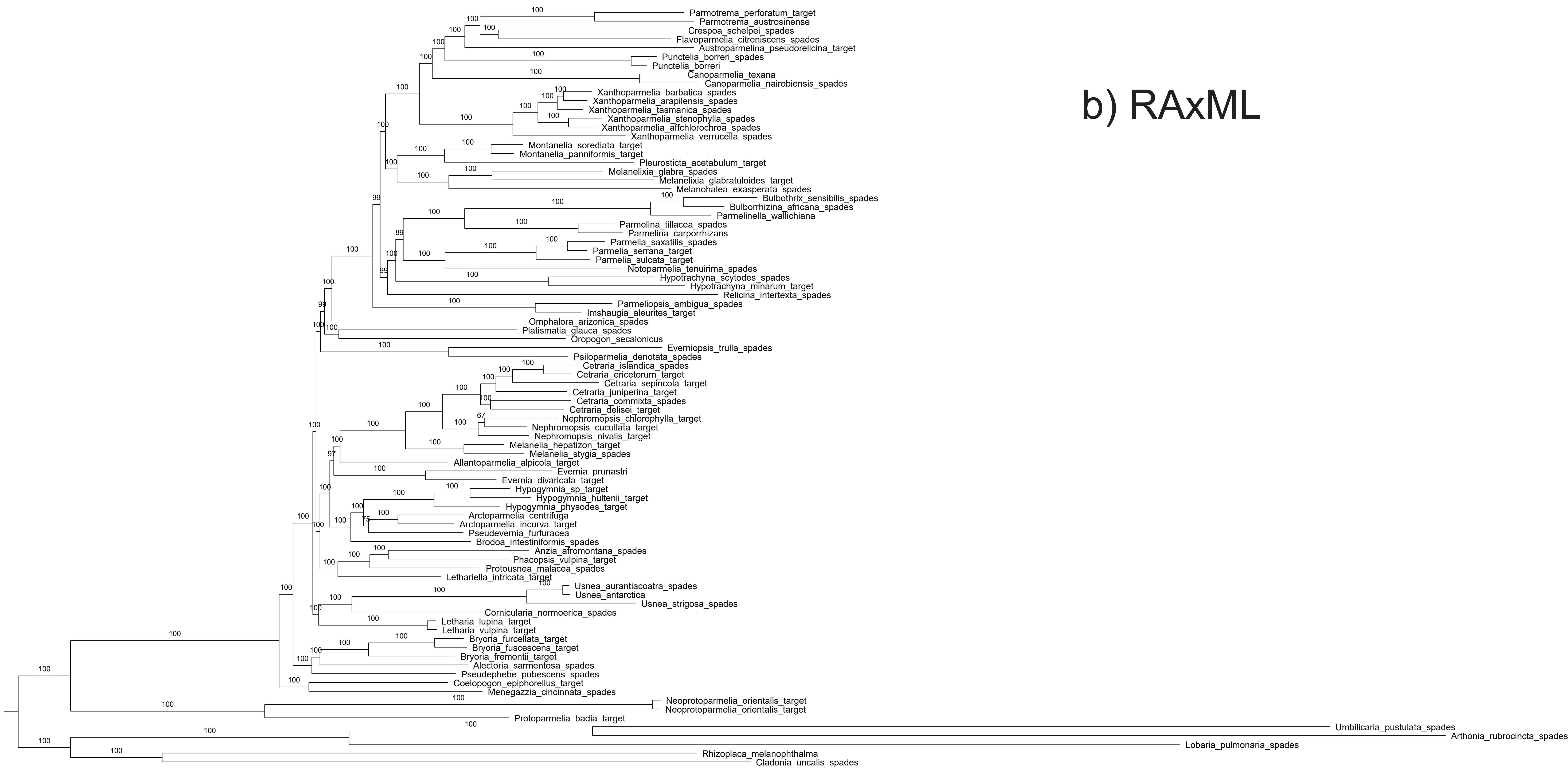

# c) MrBayes

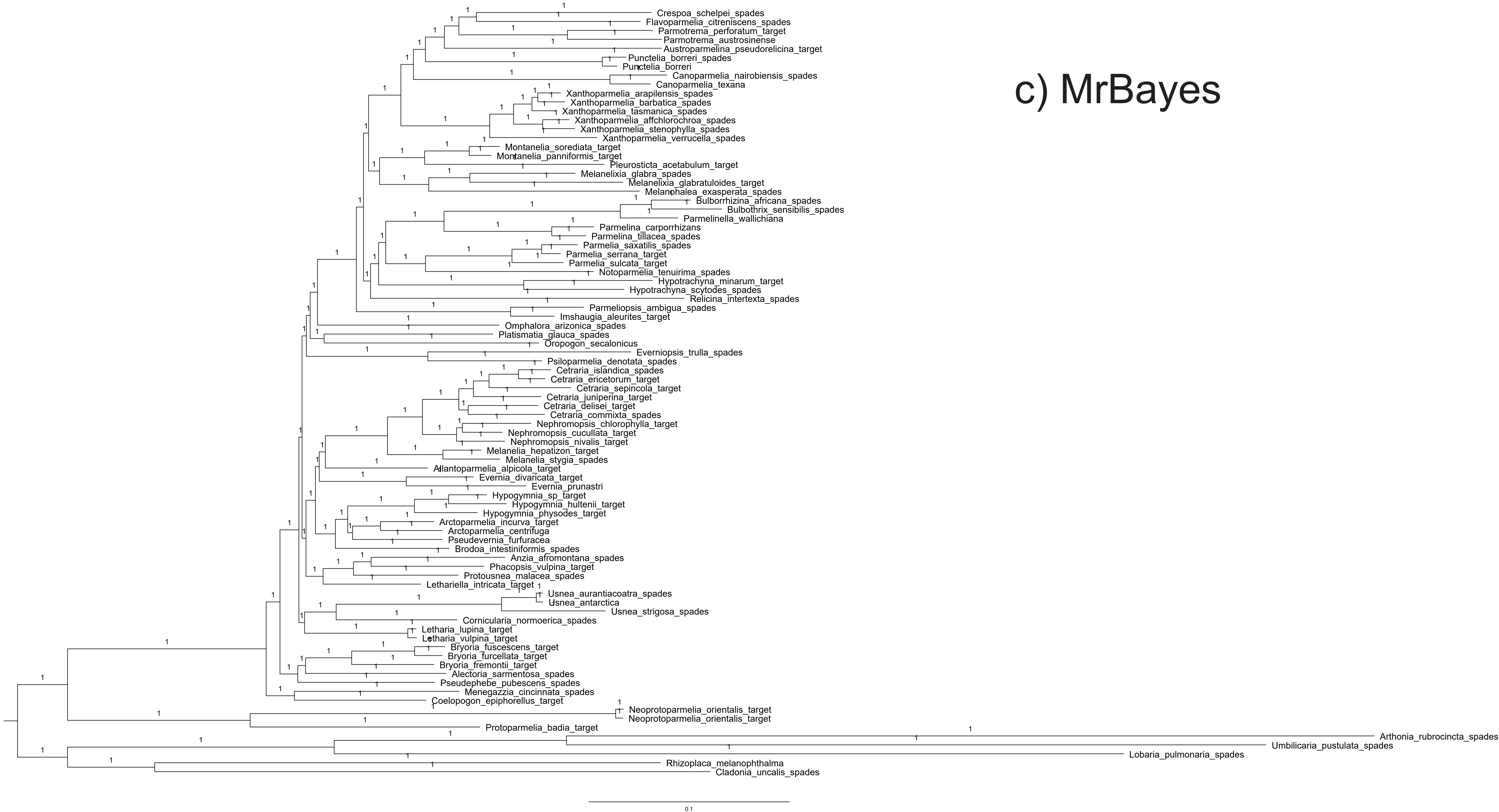

d) ASTRAL-III

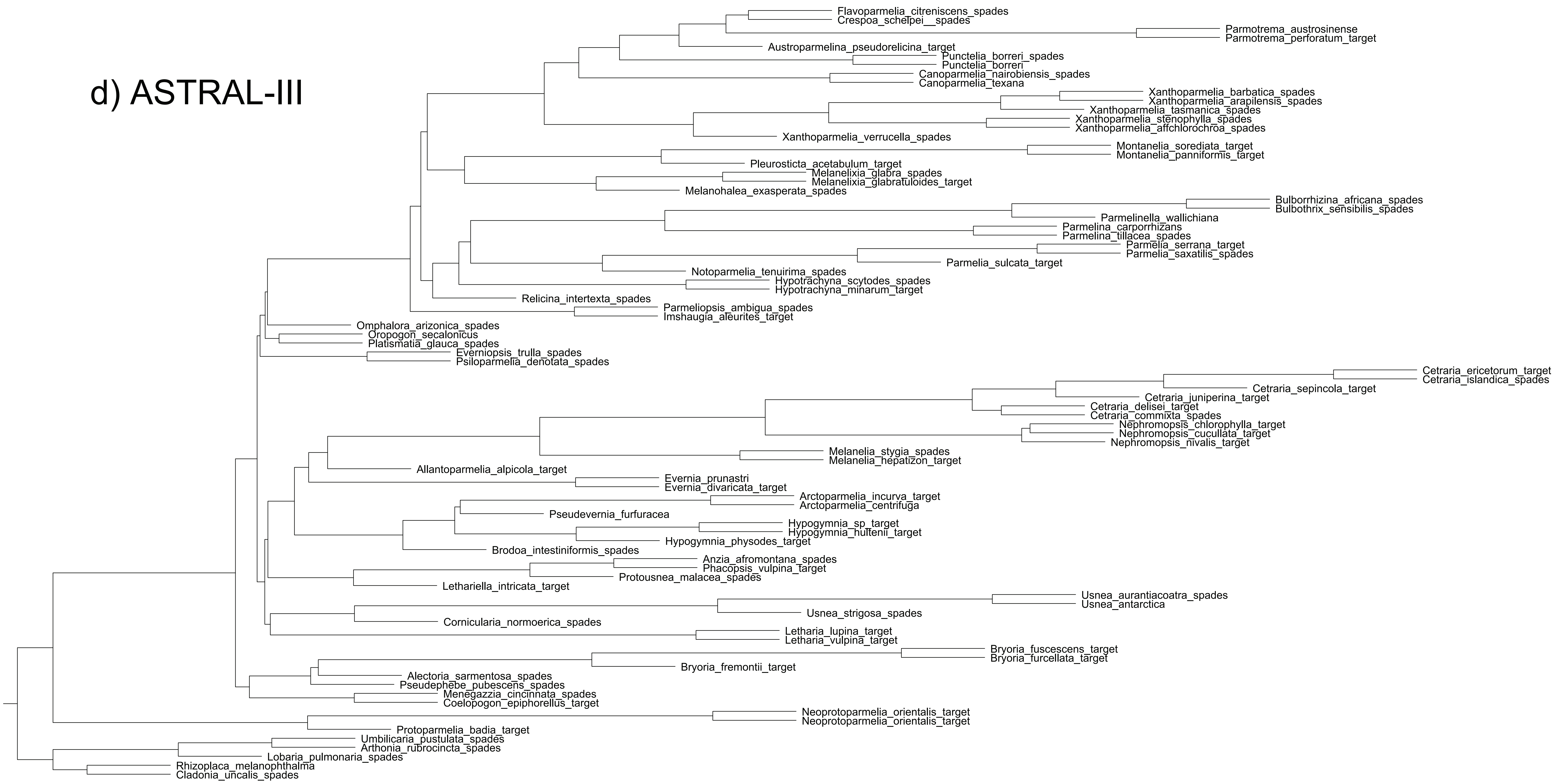

# e) IQ-TREE (fast evolving genes)

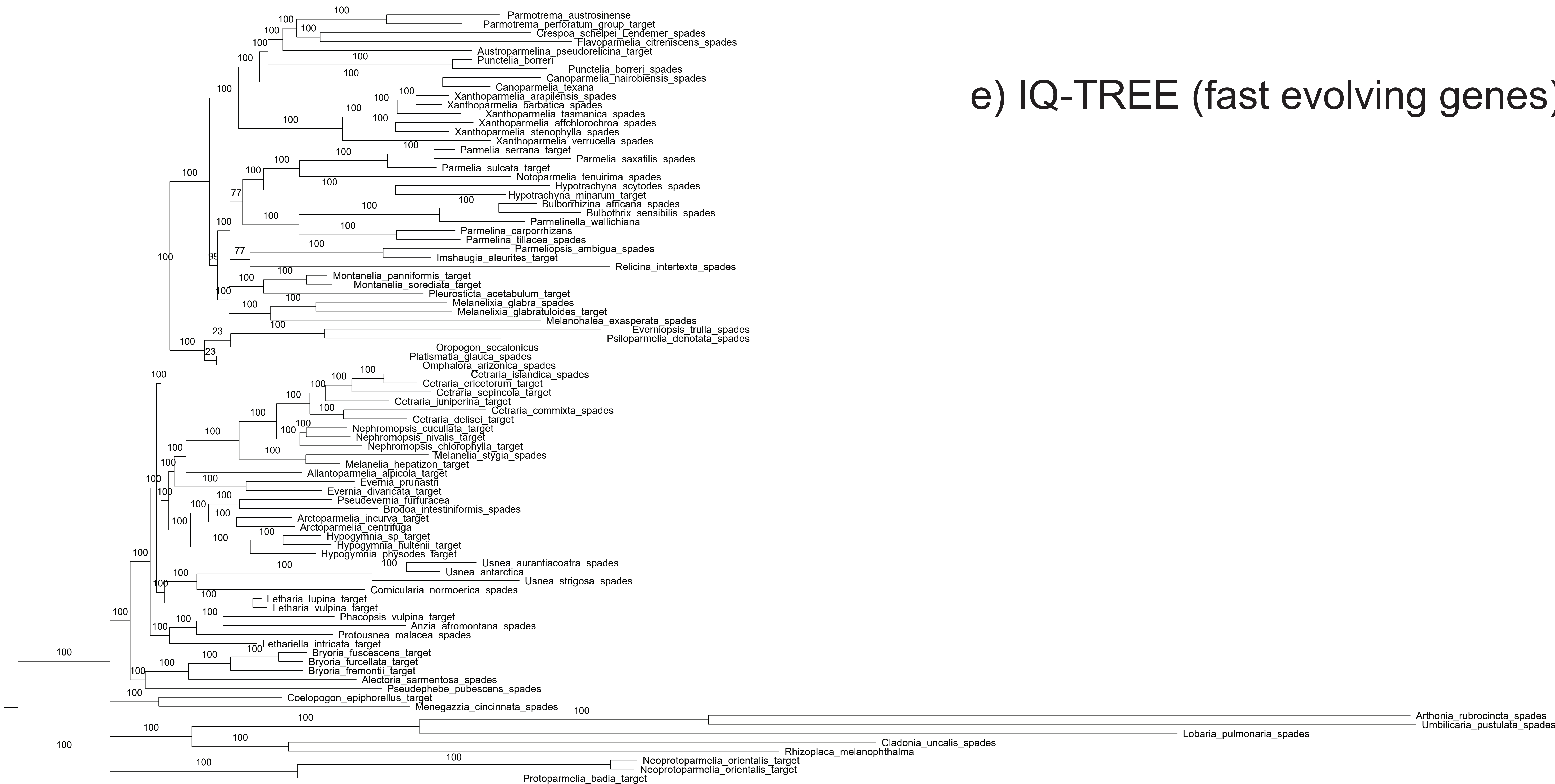

0.1

# f) RAxML (fast evolving genes)

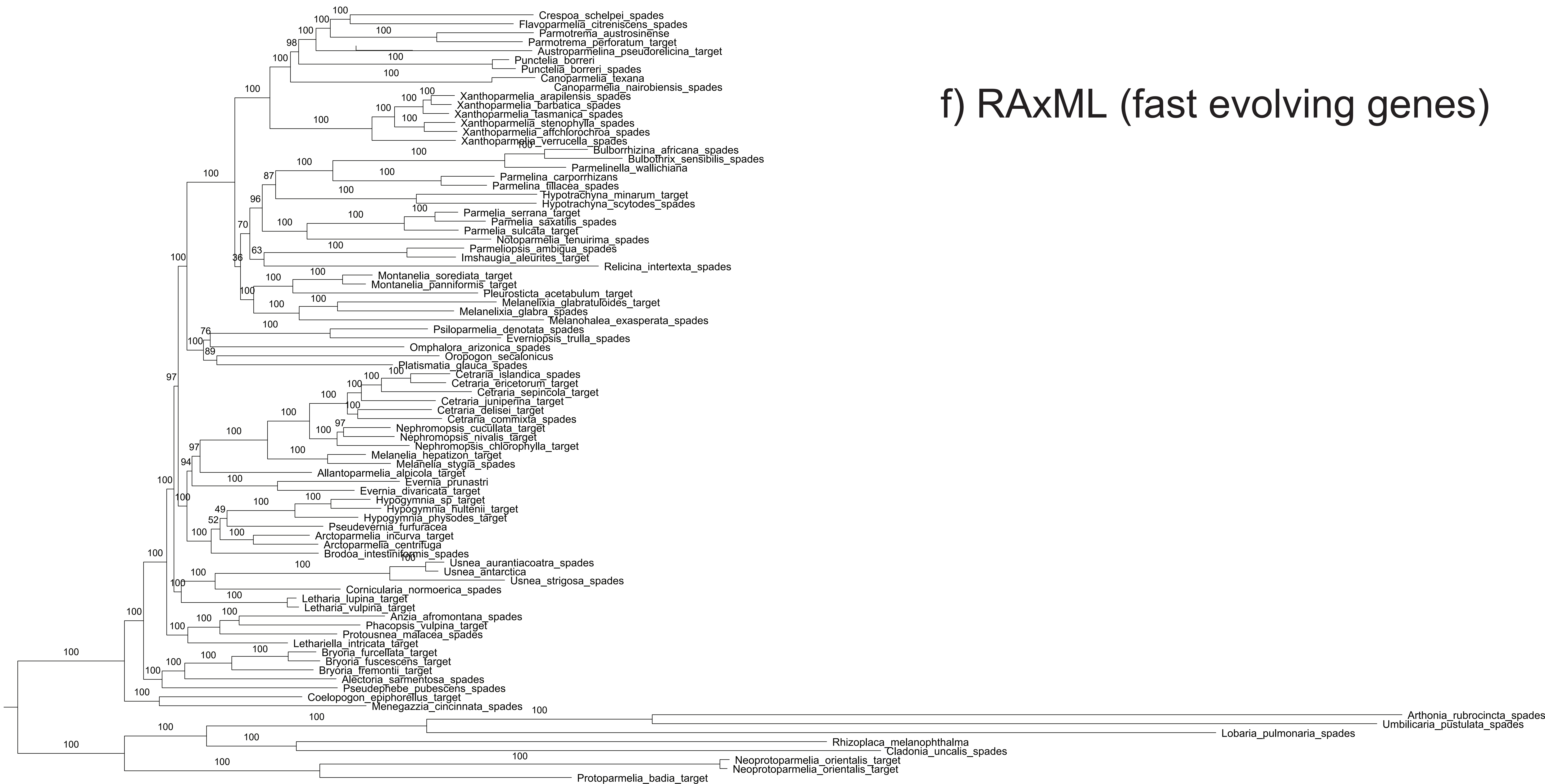

Supplement: Supplementary file 2 — Additional file 2 : Supplementary Figure 2. Phylogenetic relationships among major lineages of Parmeliaceae. Four trees were generated by Maximum Likelihood inference using a) IQ-TREE, b) RAxML, c) Bayesian interference using MrBayes, or d) a coalescent-based species tree calculation of a data set containing the 250 most phylogenetic informative genes of the target enrichment gene set. In addition, two trees were generated by Maximum Likelihood inference using e) IQ-Tree and f) RAxML of data containing the 89 fastest evolving genes. Numbers at tree branches represents IQ-TREE bootstrap, RAxML bootstrap, or MrBayes posterior probability values, respectively. The unit of branch lengths of the IQ-TREE, RAxML, and MrBayes trees is substitutions per site. Branch lengths of the tree generated by ASTRAL-III are in coalescent units. [file 43008_2020_51_MOESM2_ESM.pdf]
